# Utility of immunohistochemical markers in differentiating benign from malignant follicular-derived thyroid nodules

**DOI:** 10.1186/1746-1596-5-9

**Published:** 2010-01-26

**Authors:** Husain A Saleh, Bo Jin, John Barnwell, Opada Alzohaili

**Affiliations:** 1 Department of Pathology, Wayne State University, Sinai Grace Hospital/Detroit Medical Center, Detroit, Michigan, USA; 2 Department of Surgery, Detroit Medical Center, Detroit Medical Center, Detroit, Michigan, USA; 3 Department of Medicine, Sinai Grace Hospital/Detroit Medical Center, Detroit, Michigan, USA

## Abstract

**Background:**

Thyroid nodules are common among adults though only a small percentage is malignant, which can histologically mimic benign nodules. Accurate diagnosis of these thyroid nodules is critical for the proper clinical management.

**Methods:**

We investigated immunoexpression in 98 surgically removed benign thyroid nodules including 52 hyperplastic nodules (HN) and 46 follicular/Hurthle cell adenomas (FA), and 54 malignant tumors including 22 follicular carcinoma (FC), 20 classic papillary carcinoma (PTC), and 12 follicular variant papillary carcinoma (FVPC).

**Results:**

The staining results showed that malignant tumors express galectin-3, HBME-1, CK19 and Ret oncoprotein significantly more than benign nodules. The sensitivity of these markers for the distinction between benign and malignant lesions ranged from 83.3% to 87%. The sensitivity of two-marker panels was not significantly different. Immunoexpression was usually diffuse and strong in malignant tumors, and focal and weak in the benign lesions.

**Conclusion:**

Our findings indicate that these immunomarkers are significantly more expressed in malignant tumors compared to benign lesions and may be of additional diagnostic value when combined with routine histology.

## Introduction

Thyroid tumors are the most common endocrine tumors in the United States, and about 40% of the population between 30 and 60 years-old have thyroid nodules, most of which are benign [1]. Difficulties in the diagnosis of follicular patterned thyroid lesions on fine needle aspiration (FNA) cytology examination are well know problems, and histologic evaluation of surgically resected follicular patterned lesions can be challenging as well. One common diagnostic dilemma is encountered when an encapsulated lesion with follicular growth pattern has some but not all the nuclear features diagnostic of papillary thyroid carcinoma [2-6]. Also, follicular neoplasms are classified as benign or malignant depending on the presence or absence of capsular and/or vascular invasion. However, evaluation of these features can be challenging on histologic examination due to the presence of incomplete capsular penetration or equivocal vascular invasion, and for this reason, many end up with a general inconclusive diagnosis of "follicular lesion" [1,2].

The treatment and post-operative management of various types of thyroid nodules depends on the FNA cytologic and/or histologic diagnosis. It is estimated that only about 10% of the resected lesions are proven to be malignant [7]. Furthermore, the surgical approach of these lesions may cause anxiety and social distress, and may incur high cost for the healthcare system [8].

Currently, the standard diagnosis depends on the histomorphologic features of routine hematoxylin and eosin (H&E) stained slides, but interobserver or intraobserver disagreements in the diagnosis of follicular thyroid lesions are well known and documented [3]. For example, in a recent study, review of 200 thyroid tumors by seven Italian pathologists showed good agreement for papillary and anaplastic carcinomas, moderate for medullary and poor for follicular thyroid carcinomas.

Recent studies have focused on identifying IHC markers that can help in differentiating benign from malignant lesions, and follicular variant of papillary carcinoma from follicular carcinoma or adenoma [9-16]. Several markers have been investigated on aspiration biopsy material and histologic specimens such as CK19, galectin-3, HBME-1, CK 903, CITED1, Ret oncoprotein, CD 44, CD 57, cyclin D1 and p27. The findings were generally encouraging and promising although some studies demonstrated inconclusive or conflicting results [3,5,6,8,9,12,13,17-23].

The aim of this study was to investigate the ability of four IHC markers, individually or in combination, to distinguish between benign (non-neoplastic and neoplastic) and malignant (follicular and papillary carcinomas) thyroid lesions removed by surgical resection. The markers included galectin-3, HBME-1, CK19 and Ret oncoprotein.

## Materials and methods

A total of 152 cases of surgically resected thyroid lesions, during the period of 2004-2008, were collected from the archive of the pathology department at Sinai-Grace Hospital/Detroit Medical Center, Detroit, Michigan. The cases included 52 benign non-neoplastic lesions diagnosed as hyperplastic colloid nodules or cellular colloid nodules (HN), 46 cases of benign tumors (FA/HA) and 54 cases of malignant tumors (22 cases of FC, 20 cases of PTC and 12 cases of FVPC). Cases with equivocal features or indefinite diagnosis were excluded from the study. The diagnosis of hyperplastic colloid nodules was based on the presence of follicles containing colloid and lined by bland follicular cells with basal small round nuclei lacking crowding, overlapping or other PTC-type nuclei. Clinically, these patients had elevated serum T3 and/or T4 levels and decreased thyroid-stimulating hormone levels. The evaluation and classification of the thyroid tumors was based on WHO thyroid tumor classification published in 2004. The diagnosis of follicular adenoma was made based on the presence of encapsulated mass with homogenous follicular proliferation, lack of PTC nuclear features and absence of vascular and/or capsular invasion. Hurthle cell adenomas were defined as encapsulated tumor forming solid and small follicle growth patterns with the presence of more than 75% of the characteristic large cells with granular eosinophilic cytoplasm. The diagnosis of classic PTC was based on the presence of papillary structures with fibrovascular cores and specific nuclear features widely known as typical of papillary carcinoma. The FVPC was diagnosed based on the presence of follicular growth pattern with classic PTC-type nuclear features in at least several areas of the tumor. Follicular carcinoma was diagnosed based on the presence of follicular proliferation with complete thick capsule and full capsular penetration and/or vascular invasion, and atypical hyperchromatic nuclei that lacked features of PTC nuclei. One case of frankly invasive type of follicular carcinoma involving the parafollicular tissues was included.

All specimens were fixed in 10% zinc formalin, embedded in paraffin and 4 micron-thick sections stained with hematoxylin and eosin for routine histological examination. Immunohistochemical staining was performed using four selected markers that have been shown in recent studies to be favorably expressed in malignant thyroid tumors [9,17-23]. Table 1 shows the characteristics of the used antibodies including CK19, HBME-1, and Galectin-3 and Ret oncoprotein. All immunohistochemical stains were performed on a Ventana Benchmark automated strainer. After standard protocols for deparaffinization of the 4 micron-thick sections, and microwave antigen retrieval, the tissue sections were incubated with available commercial monoclonal antibodies diluted at 1: 100, except for Ret oncoprotein that used 1: 40 dilution, for 32 minutes. The staining was completed using a streptavidin-biotin-complex detection method. Positive controls were histiocytes for galectin-3, mesothelioma for HBME-1, skin for CK19 and a known case of PTC for Ret oncoprotein.

**Table 1:** Characteristics of the antibodies used

Antibody	Clone	Dilution	Antigen retrieval	Company
CK19	RCK108	1:100	HIER EDTA Buffer	Dako Corp
HBME	HBME-1	1:50	HIER EDTA Buffer	Dako Corp
Galectin 3	9C4	1:100	HIER Citrate Buffer	Leica Microsystems
Ret Oncoprotein	3F8	1:40	HIER EDTA Buffer	Leica Microsystems

Dako Corporation, Carpentaria, California

Leica Microsystems, Bannockburn, Illinois

CK19: cytokeratin 19

The stained slides were examined by two pathologists (HS, BJ) blindly and independently without knowing the original histologic diagnosis. A case was considered positive for a particular marker when cytoplasmic or nuclear staining of 10% or more of the lesional cells was found reactive with the antibody. The staining results were then correlated with the original histologic diagnoses and data tabulated. Table2 shows the histologic diagnosis of the cases with the staining results for each group of the thyroid lesions.

**Table 2:** Correlation of immunohistochemical staining results with histological diagnosis of thyroid lesions

Markers	Benign (98)			Malignant (54)			
	Non-Neoplastic HN 52 (%)	Neoplastic FA/HA 46 (%)	Total 98 (%)	FC 22	PTC 20	FVPC 12	Total 54 (%)
**Gal-3**	8 (15.3%)	19 (41.3%)	27(27.5%)	18 (81.8%)	18 (90%)	10 (83.3%)	46 (85.1%)
**RET**	9 (17.3%)	21 (45.6%)	30(30.6%)	16 (72.7%)	18 (90%)	11 (91.7%)	45 (83.3%)
**HBME-1**	9 (17.3%)	26 (56.5%)	35(35.7%)	18 (81.8%)	18 (90%)	11 (91.7%)	47 (87%)
**CK-19**	8(15.3%)	23 (50%)	31(31.6%)	19 (86.3%)	17 (85%)	10 (83.3%)	47 (85.1%)

HN: hyperplastic/adenomatoid nodule; FA: Follicular adenoma; HA: Hurthle cell adenoma; FC: Follicular carcinoma; PTC: Papillary thyroid carcinoma; FVPC: Follicular variant of papillary carcinoma

Interpretation and Analysis: Galectin-3 displayed cytoplasmic and nuclear staining, HBME1 staining was cytoplasmic with membranous accentuation, CK19 staining was cytoplasmic and Ret oncoprotein staining was cytoplasmic. With HBME-1 marker, only cells with distinct strong membranous staining were counted as positive reaction. A lesion was considered positive when 10% or more of the cells showed reactivity for the specific antibody. Sensitivity, specificity, positive predictive value (PPV) and negative predictive value (NPV) were calculated for each of the markers and for different combinations of these markers in the benign vs. malignant, benign non-neoplastic vs. malignant and benign neoplastic vs. malignant groups. We chose these parameters rather than the *p-value *because we were interested primarily in finding out the most sensitive and specific markers. The calculation was done using a known statistical computer software program (SPSS 10.0 for Windows; SPSS Inc. Chicago, Illinois, USA).

## Results

Table 2 shows summary of the immunohistochemical expression of each marker in each group of the thyroid lesions. In general, high percentages of the malignant tumors (FC, PTC and FVPC) demonstrated strong and diffuse reactivity for all markers, while benign lesions (both neoplastic and non-neoplastic) showed lower expression with mainly focal and weaker staining. Specifically, benign tumors (FA/HA) were less often positive than malignant, and the reactivity was less intense. Benign non-neoplastic lesions (HN) were often negative but some showed focal weak reactivity.

Table 3 shows the sensitivity, specificity, PPV and NPV in the malignant tumors (FC, PTC and FVPC) vs. all benign lesions (HN, FA/HA). All four markers had very good sensitivity ranging from 83.3% (Ret oncoprotein) to 87% (HBME-1), but the specificity was mostly moderate and ranged from 64.3% (for HBME-1) to 72.4% (for galectin-3).

**Table 3:** Sensitivity, specificity, PPV and NPV of all Benign vs. Malignant Thyroid Lesions for each of the IHC markers

Markers	Sensitivity	Specificity	PPV	NPV
Gal-3	0.852	0.724	0.630	0.899
Ret	0.833	0.694	0.600	0.883
HBME-1	0.870	0.643	0.573	0.900
CK-19	0.852	0.684	0.597	0.893

Gal-3, galectin-3; Ret, Ret oncoprotein; CK19, cytokeratin 19; PPV, positive predictive value; NPV, negative predictive value.

### Non-neoplastic lesions (HN)

**Figure 1:**
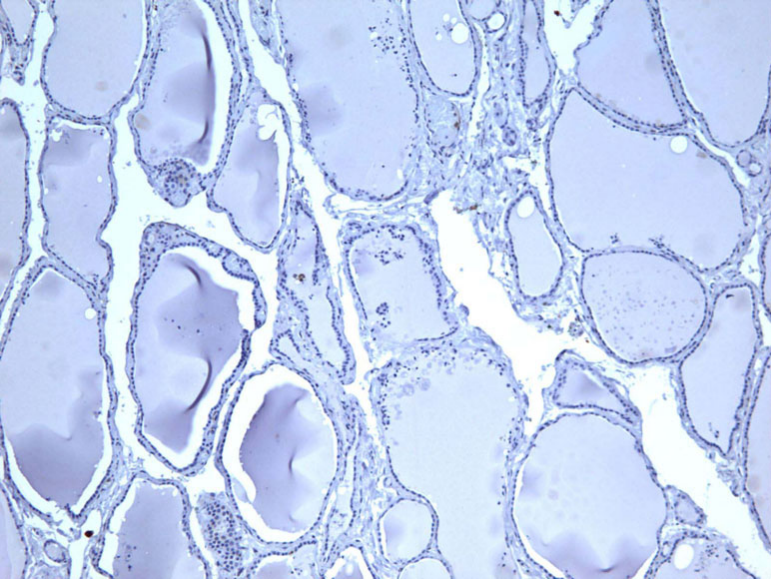
Immunohistochemical stain of hyperplastic colloid nodule shows the cells are negative for galectin-3 (100×).

**Figure 2:**
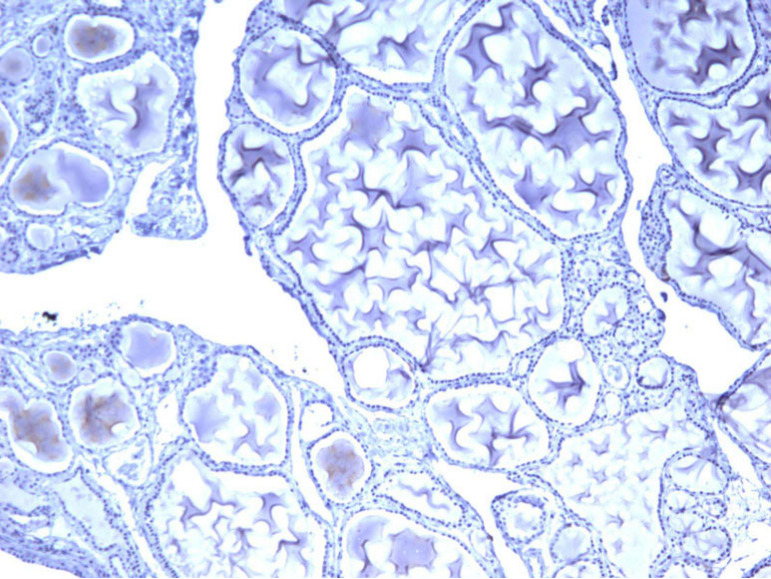
Immunohistochemical stain of hyperplastic colloid nodule shows the cells are negative for Ret oncoprotein (100×).

**Figure 3:**
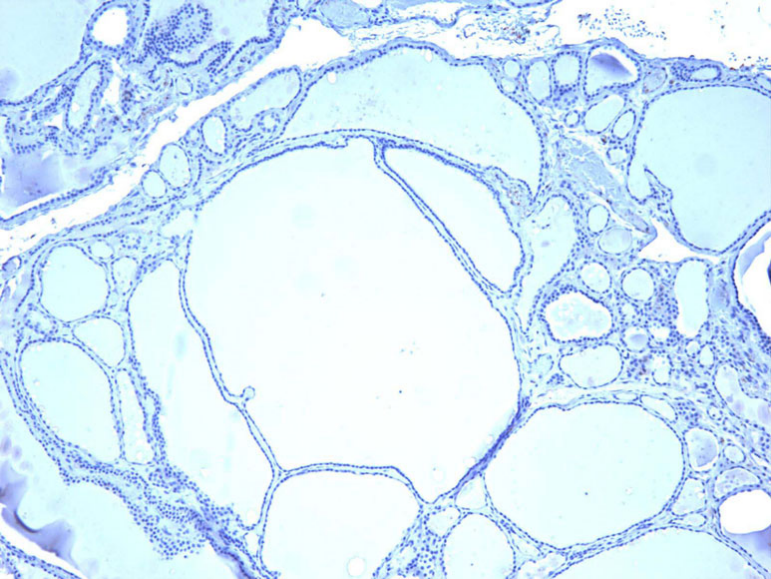
Immunohistochemical stain of hyperplastic colloid nodule shows the cells are negative, for HBME-1 (100×).

**Figure 4:**
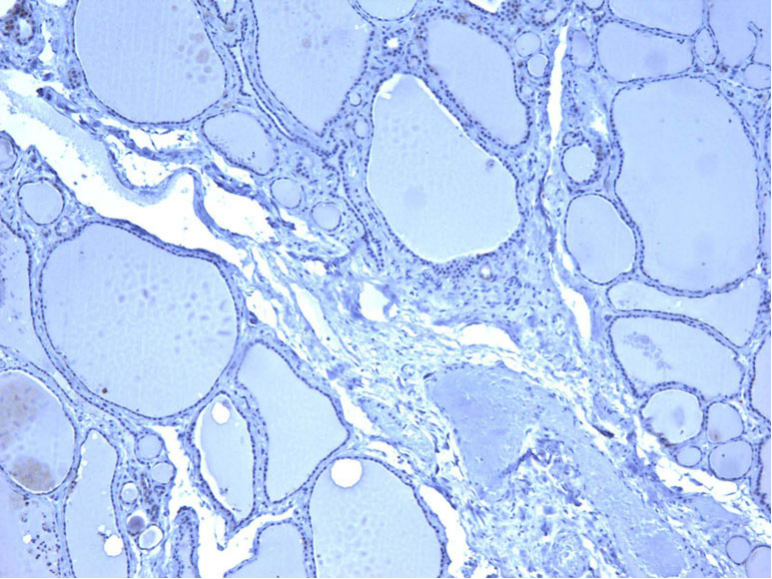
Immunohistochemical stain of hyperplastic colloid nodule shows the cells are negative for CK19 (100×).

Of the 52 cases, only 8 (15.3%) were positive for Galectin-3 and CK19, and 9 (17.3%) positive for Ret and HBME-1 (Figures 1, 2, 3, 4). Table 2 shows significant differences in the immunoexpression of all markers in the benign non-neoplastic (HN) lesions, adenomas and carcinomas ranging from mid 20%s (HN) to mid 40%s (adenomas) to mid 80%s (carcinoma).

### Adenomas

**Figure 5:**
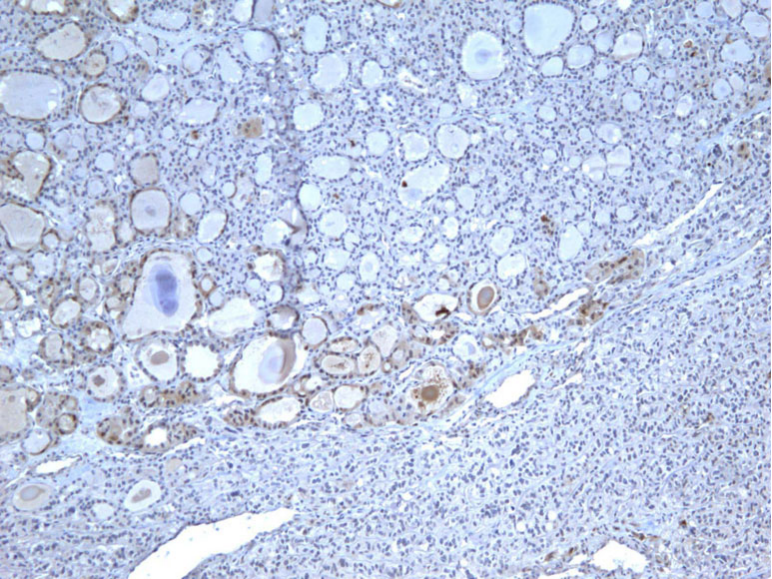
Immunohistochemical stain of follicular adenoma shows the tumor cells focally reactive for galectin-3 (100×).

**Figure 6:**
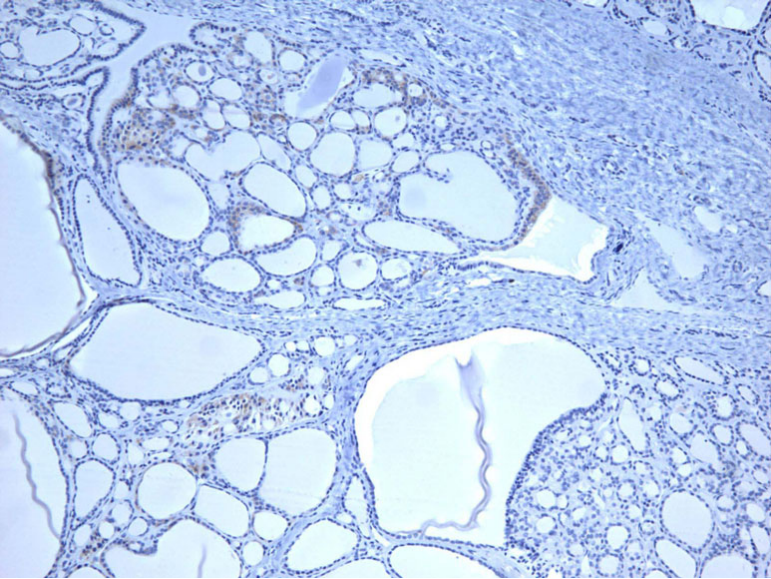
Immunohistochemical stain of follicular adenoma shows the tumor cells focally reactive for Ret oncoprotein (100×).

**Figure 7:**
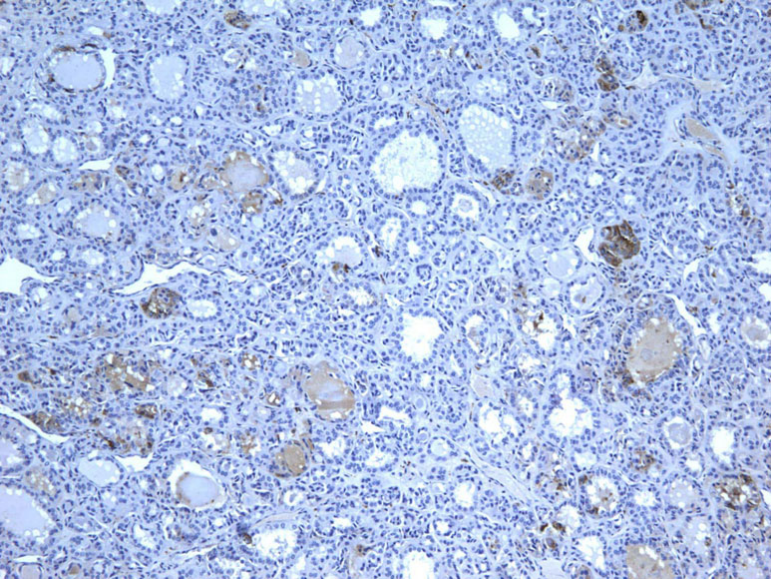
Immunohistochemical stain of follicular adenoma shows the tumor cells focally reactive for HBME-1 (100×).

**Figure 8:**
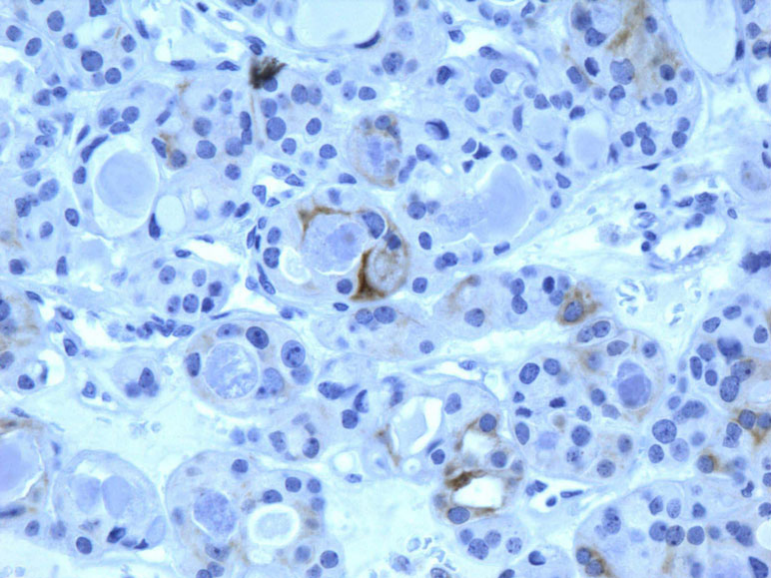
Immunohistochemical stain of follicular adenoma shows the tumor cells focally reactive CK19 (400×).

Of the 46 adenomas, 19 (41.3%) were positive for galectin-3, 21 (45.6%) for Ret, 26 (56.5%) for HBME-1, and 23 (50%) for CK19. As a group, the adenomas appeared less often positive than carcinomas for each of the four markers (Table 2) (Figures 5, 6, 7, 8). The data in Table 2 and table 4 shows that immunoexpression of all markers is much lower in the adenomas than that in carcinomas (mid 40s compared to mid 80s). The sensitivity of the immunoexpression differences of these markers between adenomas and carcinomas is high ranging from 83.3% (Ret) to 85.2% (galectin-3 and CK19) to 87% (HBME-1) (Table 4). However, the specificity is only moderate for the distinction of adenomas from carcinoma by all markers ranging from 43.5% (HBME-1) to 58.7% (galectin-3).

**Table 4:** Sensitivity, specificity, PPV and NPV of Benign Neoplastic (adenomas) vs. Malignant Thyroid Lesions

Markers	Sensitivity	Specificity	PPV	NPV
**Gal-3**	0.852	0.587	0.708	0.771
**RET**	0.833	0.543	0.682	0.735
**HBME-1**	0.870	0.435	0.644	0.741
**CK-19**	0.852	0.500	0.667	0.742

Gal-3, galectin-3; Ret, Ret oncoprotein; CK19, cytokeratin 19; PPV, positive predictive value; NPV, negative predictive value.

### Carcinomas

**Figure 9:**
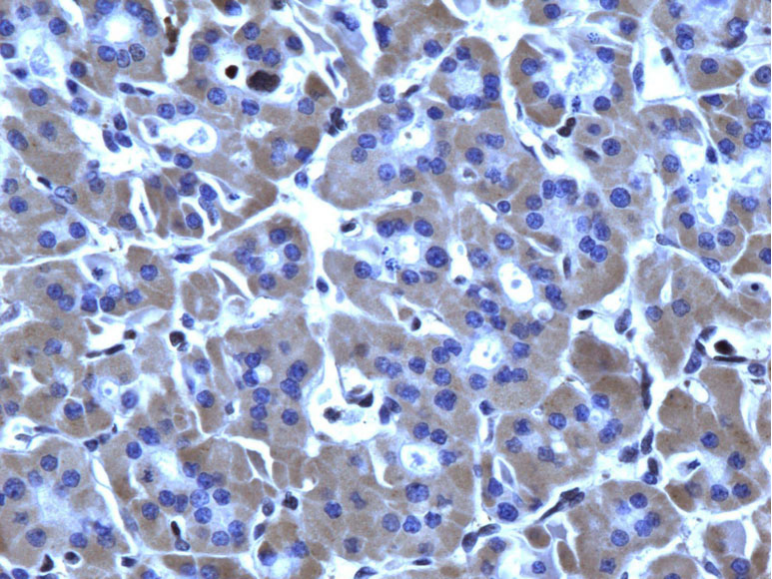
Immunohistochemical stain reveals that tumor cells in follicular carcinoma are strongly and diffusely positive for galectin-3 (400×).

**Figure 10:**
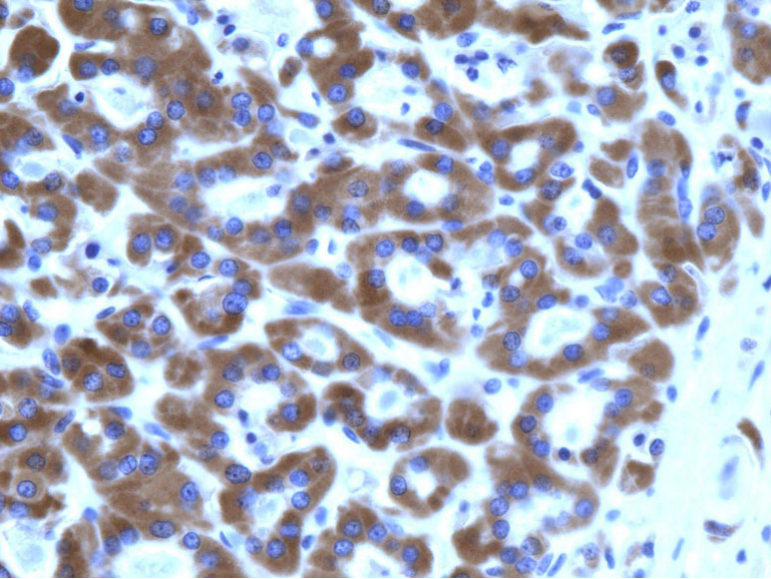
Immunohistochemical stain reveals that tumor cells in follicular carcinoma are strongly and diffusely positive for Ret oncoprotein (400×).

**Figure 11:**
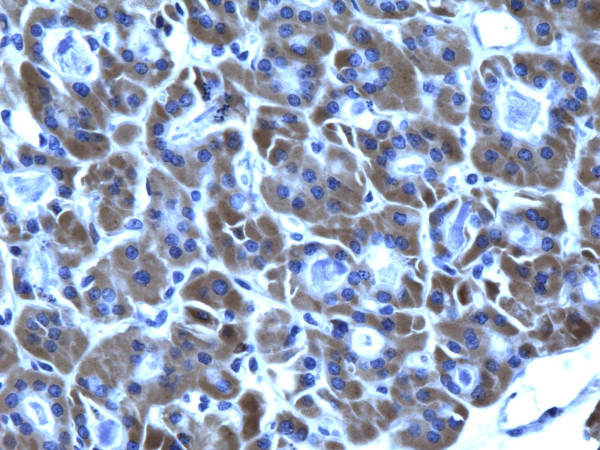
Immunohistochemical stain reveals that tumor cells in follicular carcinoma are strongly and diffusely positive for HBME-1 (400×).

**Figure 12:**
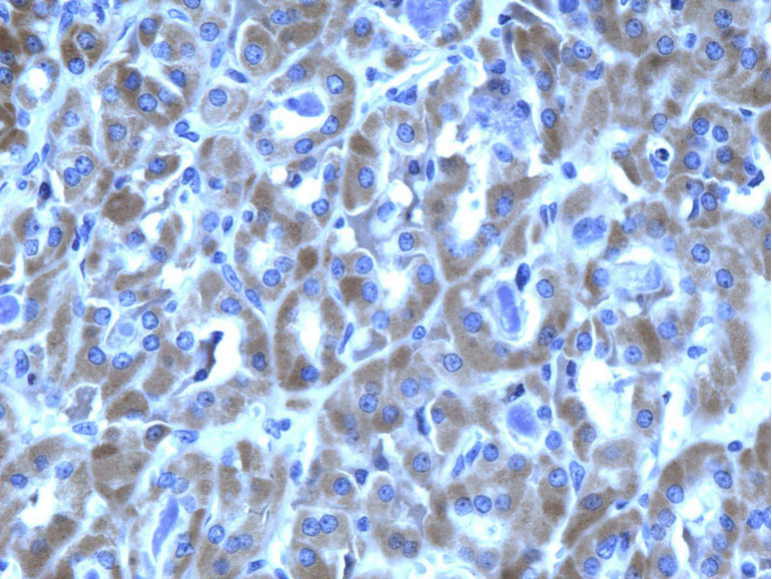
Immunohistochemical stain reveals that tumor cells in follicular carcinoma are strongly and diffusely positive for CK19 (400×).

**Figure 13:**
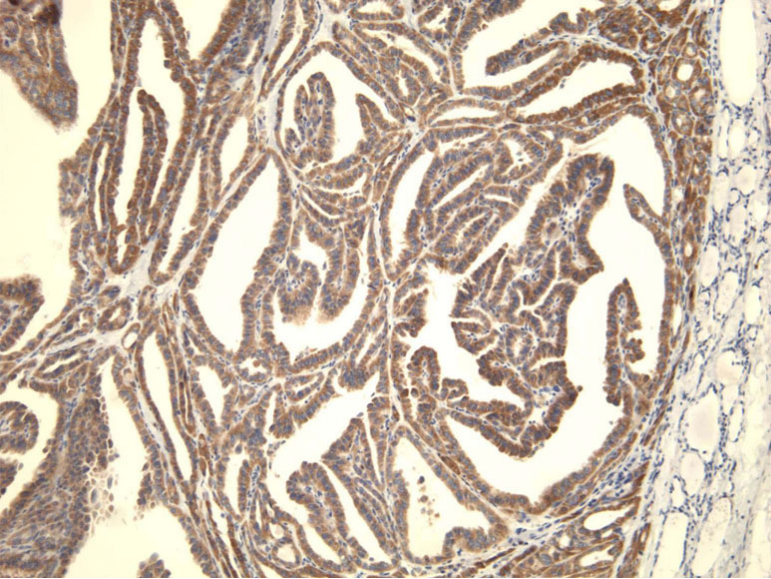
Immunohistochemical stain reveals that the tumor cells of classic papillary thyroid carcinoma are strongly and diffusely positive for galectin-3 (100×).

**Figure 14:**
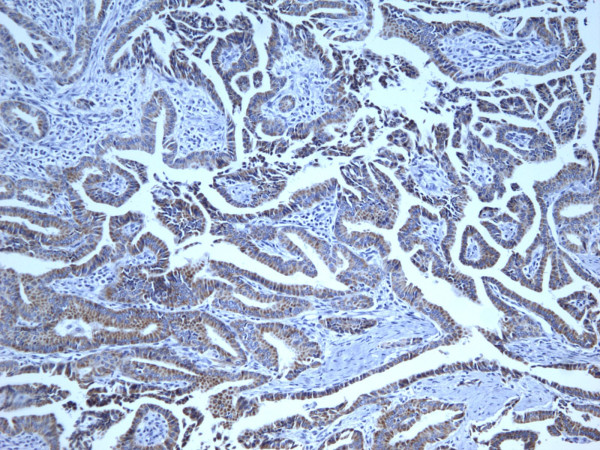
Immunohistochemical stain reveals that the tumor cells of classic papillary thyroid carcinoma are strongly and diffusely positive for Ret oncoprotein (100×).

**Figure 15:**
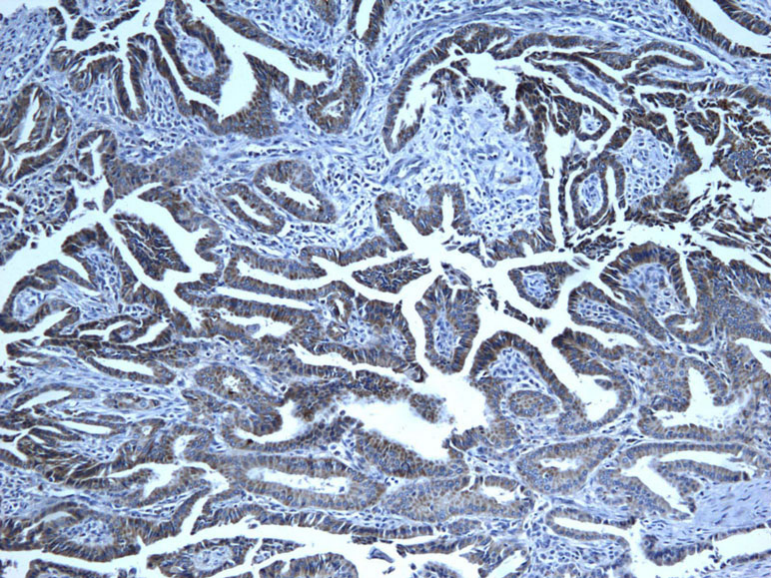
Immunohistochemical stain reveals that the tumor cells of classic papillary thyroid carcinoma are strongly and diffusely positive for HBME-1 (100×).

**Figure 16:**
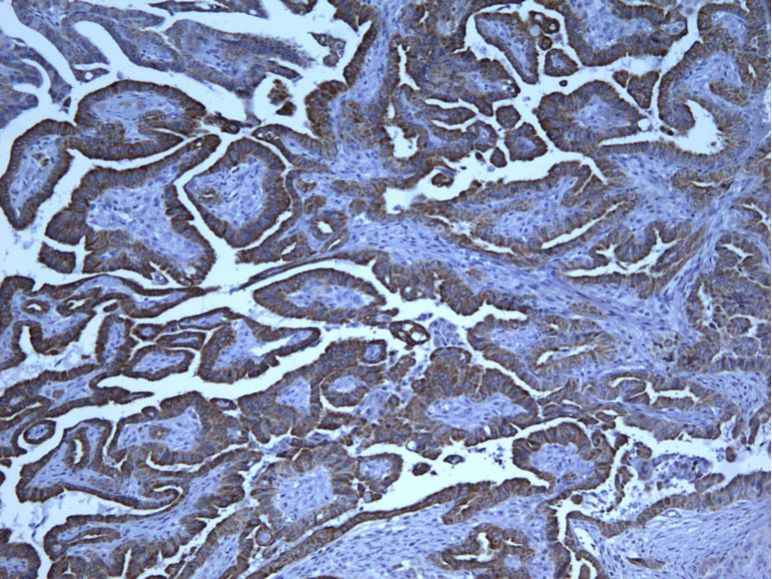
Immunohistochemical stain reveals that the tumor cells of classic papillary thyroid carcinoma are strongly and diffusely positive for CK19 (100×).

**Figure 17:**
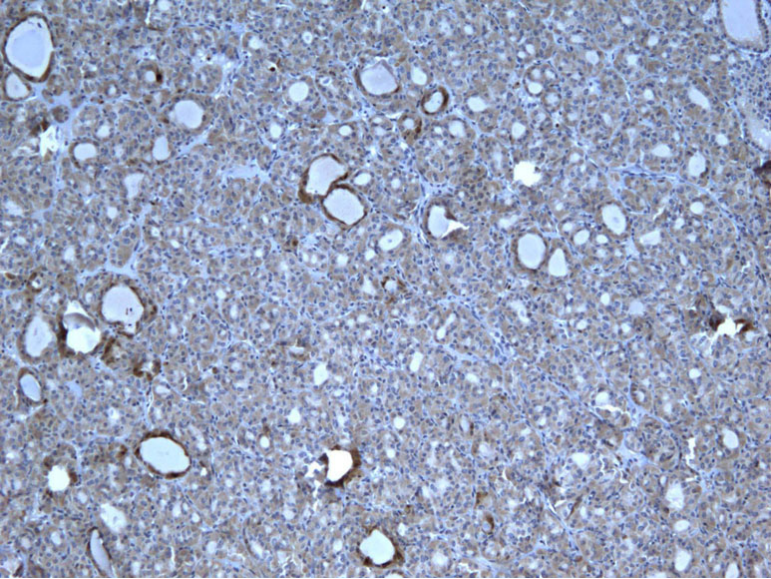
Immunohistochemical stain shows that the tumor cells of follicular variant of papillary carcinoma are strongly and diffusely positive for galectin-3 (100×).

**Figure 18:**
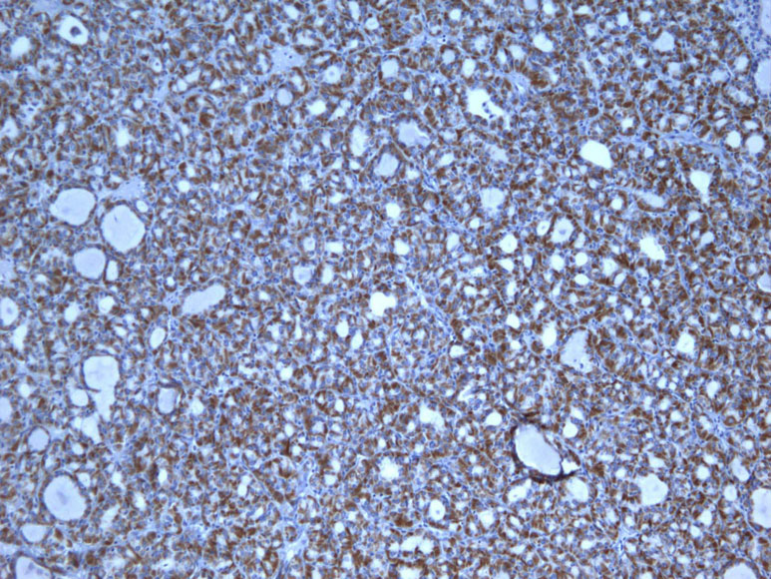
Immunohistochemical stain shows that the tumor cells of follicular variant of papillary carcinoma are strongly and diffusely positive for Ret oncoprotein (100×).

**Figure 19:**
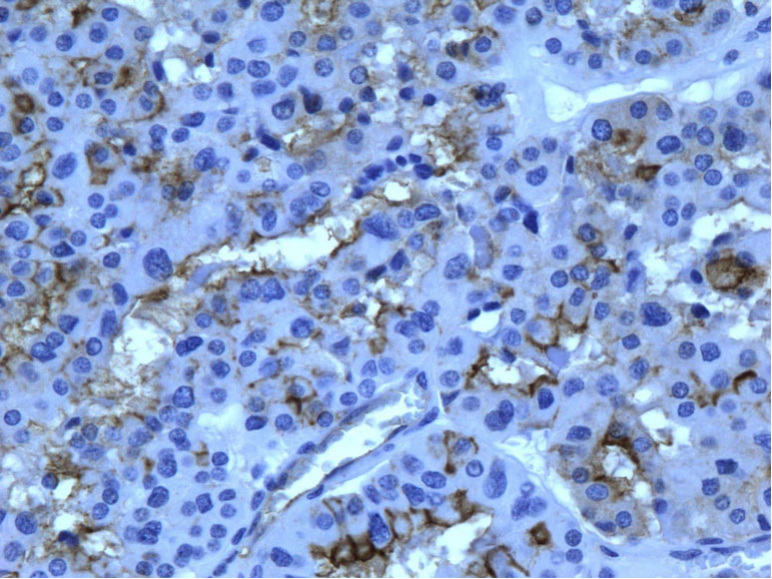
Immunohistochemical stain shows that the tumor cells of follicular variant of papillary carcinoma are strongly and diffusely positive for HBME-1 (400×).

**Figure 20:**
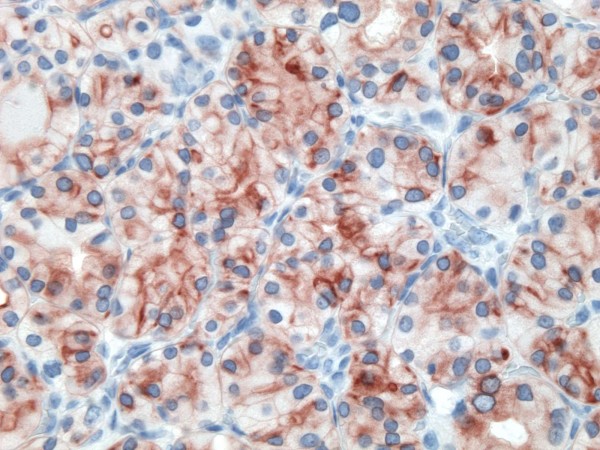
Immunohistochemical stain shows that the tumor cells of follicular variant of papillary carcinoma are strongly and diffusely positive for CK19 (400×).

of all 54 malignant tumors, 46 (85.1%) were positive for galectin-3 and CK19, 45 (83.3%) were positive for Ret and 47 (87%) positive for HBME-1. FC had high rate of reactivity especially for CK19 (19/22, 86.3%), but also for galectin-3 and HBME-1 (18/22, 81.8%) and for Ret (16/22, 72.7%) (Figures 9, 10, 11, 12). Classic PTC showed 90% (18/20) expression for galectin-3, Ret and HBME-1, while 17/20 (85%) were positive CK 19 (Figures 13, 14, 15, 16). FVPC was also highly reactive for HBME-1 and Ret (11/12, 91.6%), and for galectin-3 and CK19 (10/12, 83.3%) (Figures 17, 18, 19, 20). The data shows that all markers have high sensitivity for these subtypes of malignant thyroid tumors and therefore they do not reliably distinguish between them.

Table 5 shows that, as a group, there are significant differences in expression between non-neoplastic and malignant tumors with a sensitivity of 84.6% for galectin-3 and CK19, and 82.7% for Ret and HBME-1. The differences in percentage reactivity between HN and malignancy were as follow: 15.3% vs. 85.1% for galectin-3, 17.3% vs. 83.3% for Ret, 17.3% vs. 87% for HBME-1 and 15.3% vs. 85.1% for CK19 (Table 2).

**Table 5:** Sensitivity, specificity, PPV and NPV of Benign Non-neoplastic (HN) and Malignant Thyroid Lesions

Markers	Sensitivity	Specificity	PPV	NPV
**Gal-3**	0.846	0.587	0.698	0.771
**Ret**	0.827	0.543	0.672	0.735
**HBME-1**	0.827	0.435	0.623	0.690
**CK-19**	0.846	0.500	0.657	0.742

Gal-3, galectin-3; Ret, Ret oncoprotein; CK19, cytokeratin 19; PPV, positive predictive value; NPV, negative predictive value.

### Value of combined markers expression in benign vs. malignant lesions

Table 6 demonstrates that, as a group, the application of the panels of galectin-3+ HBME-1; galectin-3+ CK19, or HBME-1 +CK19 does not significantly increases the sensitivity or specificity for the distinction of benign vs. malignant thyroid lesions. The sensitivity of these markers combinations was as follows: 85.2% for (galectin-3+ CK19) and 86.1% for (galectin-3+HBME-1) and (HBME-1+ CK19). Furthermore, the combination panel of all three markers (galectin-3+ HBME-1+ CK19), surprisingly, did not add to the sensitivity and was only 85.8%. Similarly, the specificity of these various markers combinations, did not improve over single marker specificity and showed similar values ranging from 66.3% (HBME-1+ CK19), to 68.4% (galectin-3+ HBME-1) to 70.4% for (galectin-3+ CK19). The specificity of the three markers combination (galectin-3+ HBME-1+ CK19) was also similar at 68.4%.

**Table 6:** Comparison of sensitivity, specificity, PPV and NPV of combined markers expression in Benign vs. Malignant Thyroid Lesions

MARKER		Negative	Positive	Total		
**GAL-3 + HBME-1**	**Benign**	134	62	196	**Sensitivity**	**0.861**
	**Malignant**	15	93	108	**Specificity**	**0.684**
Total		149	155	304	**PPV**	**0.600**
					**NPV**	**0.899**
						
**GAL-3 + CK-19**	**Benign**	138	58	196	**Sensitivity**	**0.852**
	**Malignant**	16	92	108	**Specificity**	**0.704**
Total		154	150	304	**PPV**	**0.613**
					**NPV**	**0.896**
						
**HBME-1 + CK-19**	**Benign**	130	66	196	**Sensitivity**	**0.861**
	**Malignant**	15	93	108	**Specificity**	**0.663**
Total		145	159	304	**PPV**	**0.585**
					**NPV**	**0.897**
						
**GAL-3+HBME+CK-19**	**Benign**	201	93	294	**Sensitivity**	**0.858**
	**Malignant**	23	139	162	**Specificity**	**0.684**
Total		224	232	456	**PPV**	**0.599**
					**NPV**	**0.897**

Gal-3, galectin-3; Ret, Ret oncoprotein; CK19, cytokeratin 19; PPV, positive predictive value; NPV, negative predictive value.

## Discussion

The current standard in the diagnosis of thyroid lesions is by histologic examination of routine H&E stained sections. However, it is widely known that the interpretation of follicular patterned lesions can be quite difficult [1,3,5,6,22,24]. A somewhat common dilemma is encountered with encapsulated tumors showing follicular growth pattern. Presence or absence of capsular and/or vascular invasion distinguishes benign from malignant follicular tumors, but identification of this finding can be challenging due to incomplete capsular penetration, equivocal vascular invasion or technical difficulties due to processing or sectioning artifacts. Another challenging situation is encountered when some but not all of the diagnostic nuclear features of papillary carcinoma are present. Recent study by Elsheikh et al [25] and editorial review by J. Rosai [4] pointed clearly to this issue.

Thyroid nodules are fairly common clinical findings affecting approximately 40% of the population between 30 and 60 years old in the U.S.A., and thyroid cancer is the most common endocrine malignancy [8,13,26,27]. Fortunately, most of these nodules are benign tumors or hyperplastic lesions; however, it is important to identify these benign lesions for proper management and to realize maximum benefit for the patients [28]. Accurate diagnosis then is very critical for post-operative management of patients with thyroid nodules, and incorrect interpretation can lead to significant psychological and social problems, and unnecessary increase in healthcare cost [15,17-19,29,30]. Additionally, since FNA cytology in itself is not a reliable method to differentiate between benign and malignant follicular tumors or lesions, these patients usually undergo surgical resection, although only about 10% will actually have malignant tumors.

For all of the aforementioned reasons, investigators have focused during the last several years on finding molecular or IHC markers that can help in the distinction between benign and malignant lesions of the thyroid [1,9-11,14,19,21,31-33]. Identifying markers that can separate hyperplastic/adenomatous nodules from follicular tumors can be of tremendous benefits to the patients and the healthcare system[23]. As a result, many surgeries for benign lesions can be avoided and patients can be managed medically as needed [34,23]

Galectin-3 is a beta-galactoside binding polypeptide with a 31 kDa molecular weight. It is a member of the lectin family, and seems to play a significant role in a number of biological processes. It has a role in regulating cell-cell and cell-matrix interaction, adhesion, migration and damaged cell repair. It also has a role in inflammation and neoplastic transformation. Normally, it is expressed in various tissue types and tumors and appears to have a role in the invasive and metastatic potential of various tumors [15,20,27]. Kovacs et al found that IHC expression of galectin-3 may help in the differential diagnosis of solitary encapsulated follicular lesions, especially the minimally invasive follicular carcinoma [15]. Several other investigators showed that galectin-3 is very useful in distinguishing benign from malignant tumors, especially PTC, with high sensitivity and specificity [10,17-19,34]. Galectin-3 can aid in identifying FVPC, and distinguishing minimally invasive FC from FA. In our study, galectin-3 showed 85.2% sensitivity for immunoexpression distinction between carcinomas and benign nodules (positive in 27.5% of benign vs. 85.1% of malignant nodules). However, the specificity was lower at 72.4. In addition, we found that galectin-3 was somewhat more strongly and diffusely positive in PTC than in FC and FVPC, and expression in FA/HA was more focal and less intense than in malignant tumors. Also, galectin-3 expression was also detected in 8 of 52 benign non-neoplastic lesions (HN), but this was quite focal and weak. Other authors have also noted similar findings [3]. The positivity of galectin-3 in HN may be explained by the fact that follicular cells normally contain endogenous biotin that can cause false positivity. We and others also found that false positivity can be seen in cystic and inflammatory lesions. Similarly, Kovacs et al also indicated that there might be some interpretation problems caused by the observation of focal positivity in inflammatory and cystic lesions. They postulated that expression of non-neoplastic follicular cells in inflamed areas may result from cytokines secreted by the inflammatory cells or simple permeation of galectin-3 abundantly shed by lymphocytes in the neighboring follicular cells. Therefore, they suggested that IHC staining has to be evaluated in conjunction with the histological features and use of biotin-free detection system.

Some authors consider true galectin-3-positive follicular adenoma as an indication of potentially early or incipient carcinoma, in which the capsular and/or vascular invasion can not be histologically observed yet [15]. Also, some authors believe that galectin-3 immunoexpression in PTC may promote the release of tumor cells resulting in metastasis [15].

HBME-1 is a monoclonal antibody directed against an antigen on the mesothelial cell membrane. Several studies have demonstrated its preferential reactivity in malignant thyroid tumors [3,12,16,22,35]. It has been found to be reactive mostly in papillary thyroid carcinoma and some follicular carcinomas, but usually negative in follicular adenomas. Papotti et al in a study of well-differentiated thyroid tumors of uncertain malignant potential found that a diffuse and strong expression of HBME-1, and to a lesser extent galectin-3, is preferentially observed in the tumors with nuclear changes suggestive of papillary carcinoma [2]. However, they concluded that the diagnosis of these tumors should also depend on previously defined morphologic criteria. In our study, HBME-1 was expressed in 47 of total 54 thyroid carcinomas with a diagnostic sensitivity of 87%. However, it was also expressed in 9/52 (17.3%) of benign non-neoplastic lesions and in 26/46 (56.5%) of adenomas. Thus our study shows that HBME-1 is not a very good marker to distinguish adenomas from thyroid carcinomas with over half of the adenomas expressing this marker.

Several cytokeratins have been evaluated for the differential diagnosis of thyroid nodules, of which CK19 has been found to be the most useful. Studies showed that CK19 is strongly and diffusely positive in malignant thyroid tumors including classic PTC, FVPC and FC [9,10,16,31]. However, other studies showed variable results and yet others demonstrated that the CK19 expression is mostly focal and weak in FC, FA and benign hyperplastic nodules [3,22,35]. Sahoo et al, reported that 25% of their follicular adenomas had extensive immunoreactivity for CK19, and Miettinen et al also reported that 59% of their follicular carcinomas showed CK19 reactivity in more than 10% of the lesions, suggesting that CK19 expression patterns are not reliable for the distinction between papillary carcinomas and follicular neoplasms [5]. In our study, 46/54 (85.1%) of all the malignant tumors were positive for CK19 (diffusely and strongly), and 23/46 (50%) of the adenomas were also positive (but more focal and less intense). Our results showed a higher rate of CK19 reactivity in FAs than other studies, but the number of cases is relatively small for making strong conclusion. Also, we observed that positivity of CK19 in adenomas was more focal and weak than in carcinomas. In general, although most authors agree that CK19 reactivity is more frequent, diffuse and strong in papillary carcinoma, its reactivity in follicular neoplasms may limit its utility as a diagnostic marker [22].

The Ret gene is located on chromosome 10 q and encodes a tyrosine kinase transmembrane receptor [12]. It is typically absent in the normal thyroid follicular cells; however, gene rearrangement occurs in most PTCs. This oncogene is believed to be specific to PTC and encodes an oncoprotein product that contains the cytoplasmic portion of Ret gene. Therefore, some investigators believe that IHC expression of Ret oncoprotein is a reliable marker for PTC [12,33]. Cheung et al showed immunoexpression of Ret in 78% of PTC, 63% of FVPC and 57% of Hurthle cell carcinoma, while all benign nodules were non-immunoreactive for Ret. In our study, Ret was positive in 18/20 cases of PTC, in 45/54 (83.3%) of all carcinomas, and in 30/98 (30.6%) benign lesions. Rossie et al found that Ret had focal or moderate immunoreactivity in benign lesions while it showed prevalent cytoplasmic expression in classic papillary cacrcinoma and its variants. The general conclusion among researchers is that diffuse immunoexpression represents a good supportive evidence for the diagnosis of papillary carcinomas; however, focal staining is often found in other lesions including benign nodules. Perhaps, a more important finding is that immunoreactivity of a panel that includes Ret, HBME-1 and CK19 is very specific for papillary carcinoma. Cheung et al concluded that HBME-1 positivity indicates malignancy, whereas diffuse CK19 and/or Ret positivity confirm papillary differentiation [12].

Our study shows some different findings in staining reactions with other studies. We believe that some of the differences are due to various factors including antibodies used, dilution and antigen retrieval methods, type of tissue fixative used and time of fixation. Some authors have also investigated the method of IHC staining and found that galectin-3 reaction may be impacted by biotin-like activity produced by some thyroid lesions [14]. They recommended performing galectin-3 IHC staining of thyroid lesions using biotin-free detection system. We believe there is a need to standardize fixation, antibody specifics, detection systems and IHC processes to be more able to compare results of various studies.

Many studies have evaluated the immunoexpression of a single marker such as galectin-3 or CK19 in the investigation of various thyroid lesions. In this study, we aimed at evaluating multiple markers to compare their sensitivity and usefulness, and to find out if a specific combination of the evaluated markers (galectin-3, HBME-1, CK-19 and Ret oncoprotein) can be of additional value in discriminating between benign and malignant thyroid lesions. We found that these markers combinations of galectin-3+HBME-1; galectin-3+CK19 or HBME-1+ CK19 do not improve the sensitivity or specificity for the distinction between benign and malignant thyroid lesions. Furthermore, using a combination panel of three markers (galectin-3+ HBME-1+ CK19) also did not increase the sensitivity or specificity for the distinction between benign and malignant thyroid lesions. On the other hand, other investigators have found that panels of various combinations of IHC markers can add to the value of diagnosing malignant thyroid tumors and discriminating them from benign tumors and non-neoplastic lesions [23]. For example, De Matos et al found that a panel of galectin-3, HBME-1 and CK19 is useful in differentiating the follicular patterned lesions, and specifically distinguishing FVTC from FC or FA [35]. Cheung et al recommended using a panel of CK19, HBME-1 and Ret as a useful means for diagnosing papillary carcinoma; whereas Rossie et al concluded that a panel of only HBME-1 and galectin-3 can correctly diagnose classic and variants of papillary carcinomas.

In summary, immunoexpression of galectin-3, CK19 and HBME-1 is an important supplementary test in the diagnosis of thyroid neoplasms, albeit it does not replace the conventional histomorphological examination. We found that these markers have somewhat similar sensitivity and specificity of immunoexpression in thyroid malignancy. We also found that combination panels of 2 or 3 of these markers (galectin-3+ HBME-1; galectin-3+ CK19; HBME-1+ CK19 or galectin-3+ HBME-1+ CK19) do not significantly improves the sensitivity or specificity of immunoexpression in malignant tumors. However, using a panel of two markers is advised to avoid instances of technical problems or processing issues. Therefore, we recommend using these panels as useful means to increases the likelihood of detecting malignant tumors. This practical low cost IHC test of commercially available markers can help to optimize the management of patients with thyroid nodules and reduce unnecessary surgical resection of benign nodules. Nonetheless, there are still questions to answer and additional studies are needed toward the quest of identifying useful markers for differentiating benign from malignant thyroid nodules.

## Ethics

There is no ethical approval or consent required.

## Competing interests

The authors declare that they have no competing interests.

## Authors' contributions

HS: study design, pathology interpretation and constructing paper, BJ: pathology interpretation, JB and OA: clinical data collection. All authors read and approved the final manuscript.
